# Females smell differently: characteristics and significance of the most common olfactory sensilla of female silkmoths

**DOI:** 10.1098/rspb.2023.2578

**Published:** 2024-01-17

**Authors:** Elisa Schuh, Sina Cassau, Ahmed R. Ismaieel, Regina Stieber, Jürgen Krieger, Bill S. Hansson, Silke Sachse, Sonja Bisch-Knaden

**Affiliations:** ^1^ Department of Evolutionary Neuroethology, Max Planck Institute for Chemical Ecology, Hans-Knoell-Straße 8, 07745 Jena, Germany; ^2^ Research Group Olfactory Coding, Max Planck Institute for Chemical Ecology, Hans-Knoell-Straße 8, 07745 Jena, Germany; ^3^ Institute of Biology/Zoology, Department of Animal Physiology, Martin Luther University Halle-Wittenberg, Hoher Weg 8, 06120 Halle (Saale), Germany; ^4^ Entomology Department, Faculty of Science, Ain Shams University, Abbassia, Cairo 11566, Egypt

**Keywords:** olfactory, sensilla, *Bombyx mori*, pheromone, oviposition, receptor

## Abstract

In the silkmoth *Bombyx mori*, the role of male sensilla trichodea in pheromone detection is well established. Here we study the corresponding female sensilla, which contain two olfactory sensory neurons (OSNs) and come in two lengths, each representing a single physiological type. Only OSNs in medium trichoids respond to the scent of mulberry, the silkworm's exclusive host plant, and are more sensitive in mated females, suggesting a role in oviposition. In long trichoids, one OSN is tuned to (+)-linalool and the other to benzaldehyde and isovaleric acid, both odours emitted by silkworm faeces. While the significance of (+)-linalool detection remains unclear, isovaleric acid repels mated females and may therefore play a role in avoiding crowded oviposition sites. When we examined the underlying molecular components of neurons in female trichoids, we found non-canonical co-expression of *Ir8a*, the co-receptor for acid responses, and *ORco*, the co-receptor of odorant receptors, in long trichoids, and the unexpected expression of a specific odorant receptor in both trichoid sensillum types. In addition to elucidating the function of female trichoids, our results suggest that some accepted organizational principles of the insect olfactory system may not apply to the predominant sensilla on the antenna of female *B. mori*.

## 1. Introduction

The antenna of male moths is dedicated to the detection of female sex pheromones, because most of the hair-like olfactory sensilla that cover it are of one type—long sensilla trichodea—and house pheromone-specific olfactory sensory neurons (OSNs). In many moth species, females lack this sensillum type, resulting in a striking sexual dimorphism of the antenna [1,2]. In the domesticated silkmoth *Bombyx mori*, however, the antennae of both sexes are morphologically similar (electronic supplementary material, figure S1) and possess identical sets of olfactory sensilla, including two types of sensilla trichodea (long and medium) [3]. Both trichoid types together make up the largest proportion of antennal olfactory sensilla in both male (77%) and female (66%) silkmoths [3,4]. Female *B. mori* are anosmic to their own pheromone [2]. We therefore asked whether female trichoids might be dedicated to the detection of mulberry leaves, the silkworm's exclusive food, since this plant bouquet is particularly important for females when searching for an oviposition site. Female trichoids may also be involved in other behavioural contexts, such as pheromonal communication.

Each long trichoid and most of the medium trichoids of both male and female *B. mori* house two OSNs [5]. While OSNs in male long trichoids are narrowly tuned to the female pheromone bombykol and the behavioural antagonist bombykal, respectively [6], the two OSNs in female long trichoids have been described to be best activated by the terpenes (±)-linalool and α-terpineol (‘terpene cell’, A-neuron with larger spike amplitude), or by benzoic acid and benzaldehyde (‘benzoic acid cell’, B-neuron with smaller spike amplitude) [2,7,8]. However, these previous physiological studies lacked screening of female long trichoids with a wider range of odorants; and the receptive range of OSNs in medium trichoids was not investigated. We therefore recorded the response of OSNs in both long and medium trichoids to 76 monomolecular odorants from different chemical classes. We also tested complex mixtures emitted from natural sources of potential importance to female silkmoths, such as the headspace of mulberry leaves, which contains (±)-linalool among other compounds [9]. In addition to being released from plants, terpenes, i.e. possible ligands for the ‘terpene cell’, are widespread body volatiles produced by various insects, e.g. butterflies [10], and may play a role in precopulatory behaviour [11]. We therefore included headspace from female and male silkmoths as stimuli for female sensilla trichodea. We also re-examined the response of the ‘benzoic acid cell’ to stimulation with meconium [12], the liquid waste excreted by moths during eclosion, and tested the headspace of silkworm faeces, which may play a role in the choice of oviposition sites, as has been found in the hawkmoth *Manduca sexta* [13]. Although domesticated silkmoths cannot fly, *B. mori* males immediately begin to fan their wings upon detecting bombykol and run towards the pheromone-releasing female [14,15], making them a useful model organism for studying odour-guided behaviour. By contrast, *B. mori* females are immobile for most of their lives. Therefore, the behavioural relevance of female trichoid ligands is unknown.

The molecular basis of odour-evoked responses is in olfactory receptor genes expressed by OSNs. There are two major families of olfactory receptors in insects, odorant receptors (ORs) and ionotropic receptors (IRs) [16–18], which differ in their molecular receptive range. Members of the larger family of ORs are co-expressed with their obligate OR-co-receptor ORco [19], and detect a wide range of chemically diverse molecules, including insect-emitted volatiles that act as pheromones and plant-emitted volatiles such as terpenes. IRs, on the other hand, have a more restricted spectrum, but also require the co-expression of co-receptors that specify the function of a given IR: IRs co-expressed with *Ir8a* are tuned to acids, while IRs co-expressed with *Ir25a* and *Ir76b* detect amines and aldehydes [20–22]. Typically, each OSN expresses only one olfactory receptor, with neurons in sensilla trichodea and basiconica expressing ORs and neurons in sensilla coeloconica expressing IRs, i.e. ORs and IRs are mostly expressed in separate chemosensory subsystems [16,23,24]. So far, 71 ORs and 30 IRs have been identified in *B. mori* [9,25,26]. Three ORs (*BmorOr19*, *45* and *47*) are mainly present in the female antenna [27], and *BmorOr19*-expressing cells co-localize with *BmorOr45*-expressing cells in the same sensillum [28]. Furthermore, *BmorOr19* is tuned to detect (±)-linalool, whereas both *BmorOr45* and *47* are best activated by benzoic acid and benzaldehyde out of 26 odours tested [28], elucidating *BmorOr19* as the receptor expressed in the ‘terpene cell’ and *BmorOr45* and/or *47* as the receptor(s) expressed in the ‘benzoic acid cell’. However, the expression of these ORs has not been assigned to a specific sensillum type and other candidate receptors have not been tested, leaving our knowledge of the molecular basis of olfactory responses by OSNs in trichoid sensilla incomplete.

## 2. Results

### (a) Receptive range of long sensilla trichodea

**Figure 1.**
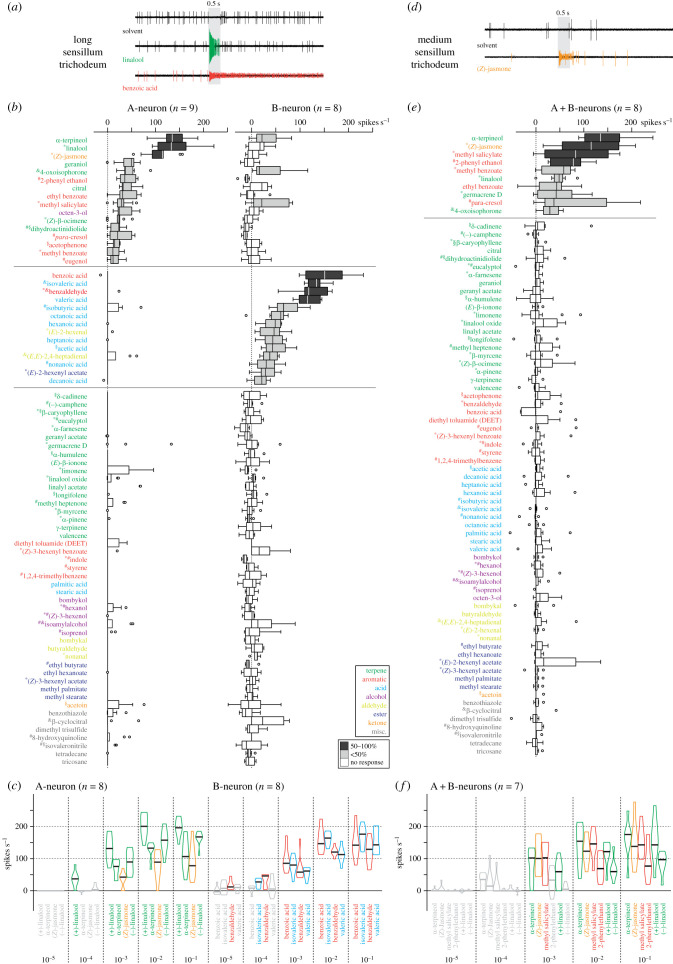
Response profile of female sensilla trichodea. (*a*) Example recordings from long sensillum trichodeum stimulated with the solvent hexane, (±)-linalool (green, large spikes, A-neuron) and benzoic acid (red, small spikes, B-neuron); diluted 10^−2^ in hexane; *grey background*, odour exposure. (*b*) Responses of A-neurons (left) and B-neurons (right) in long trichoids. *Boxplots*, net, solvent-subtracted maximum spike frequencies; *filled boxplots*, data differ from zero (*p* < 0.05, Wilcoxon rank sum test); *shades of grey*, strength of response (dark grey, ≥half maximal response; light grey, <half maximal response). Stimuli sorted according to median response first in A-neurons, then in B-neurons; stimuli eliciting no response in either neuron sorted alphabetically according to chemical class. *Symbols next to odour names*, presence in headspace from mulberry leaves (*), silkmoths (§), their meconium (#), or silkworm faeces (&); for chemical composition of headspaces see electronic supplementary material, figure S1. (*c*) Dose–response experiments with best ligands of long trichoids; *violin plots*, net maximum spike frequencies (no. spikes s^−1^) to five (v/v) odour concentrations corresponding to 60 ng, 600 ng, 6 µg, 60 µg, 600 µg of pure substance on filter paper; *horizontal line*, median; *grey violin plots*, data not different from zero (*p* > 0.05, Wilcoxon rank sum test). (*d*) Example recordings from medium sensillum trichodeum upon stimulation with hexane and (*Z*)-jasmone (orange, A + B-neurons). (*e*) Pooled responses of A + B-neurons in medium trichoids. (*f*) Dose–response experiments with best ligands of medium trichoids and both enantiomers of linalool.

Using single sensillum recordings (SSR), we found two physiological types of sensilla trichodea that occurred in similar numbers on the female antenna, corresponding to long and medium trichoids [3,5]. The two OSNs in one of the types differed in spike amplitude and spontaneous activation frequency, and appeared to be identical to previously described OSNs in long trichoids with comparable characteristics [12]. These neurons not only had different spike amplitudes and spontaneous activity (figure 1*a*), but also had almost exclusive response spectra (figure 1*b*). Of the 76 monomolecular stimuli tested, A-neurons responded strongly (≥50% of the maximum response) to only three odours: (±)-linalool and α-terpineol, as earlier reported [2], and (*Z*)-jasmone, a previously unknown ligand for A-neurons. Thirteen other odours elicited a significant but smaller response (less than 50% of the maximum response). Although (±)-linalool and (*Z*)-jasmone are among the volatiles emitted by mulberry leaves, A-neurons did not respond to mulberry headspace (table 1), probably owing to the low concentration of these odours (electronic supplementary material, figure S2*a*). Similarly, neither silkmoth headspace (containing mainly terpenes; electronic supplementary material, figure S2*b*) nor their meconium (containing molecules of different chemical classes such as terpenes, aromatics, alcohols and acids; electronic supplementary material, figure S2*c*) elicited a response (table 1). Only headspace from silkworm faeces (electronic supplementary material, figure S2*d*) containing the minor ligands 4-oxoisophorone and dihydroactinidiolide activated A-neurons.

**Table 1.** Activation of OSNs in female sensilla trichodea by natural mixtures. ‘X’ depicts difference of solvent-subtracted maximum spike frequency from zero, *p* < 0.05, Wilcoxon rank sum test.

odour source	long trichoids (A)	long trichoids (B)	medium trichoids (A + B)
mulberry leaves	−	−	x
silkmoth body odour (♀/♂)	−/−	−/−	−/−
meconium (♀/♂)	−/−	x/x	−/−
silkworm faeces	x	x	−

B-neurons showed a similar narrow tuning (figure 1*b*), responding strongly to only four odours: benzoic acid and benzaldehyde, confirming previous results [7,8], and the newly identified ligands isovaleric acid and valeric acid. Thirteen other odours, mostly carboxylic acids, were minor ligands. Consistent with a previous report [12], silkmoth meconium activated B-neurons (table 1), although none of their best ligands was present in the mixture. However, some acids were released from the meconium samples (electronic supplementary material, figure S2*c*) and may have led to the observed activation of B-neurons in long trichoids. In addition, B-neurons responded to the headspace of silkworm faeces (figure 1*d* and table 1), which contained two of the best ligands (benzaldehyde, isovaleric acid) and two other minor ligands.

The pheromones bombykol and bombykal did not activate either OSN in female long trichoids, as had been found in previous studies [2].

The high specificity of OSNs housed in female long trichoids was illustrated by high values for lifetime sparseness (*S* = 0.89 (A-neuron), *S* = 0.82 (B-neuron); electronic supplementary material, figure S3*a*), a measure of selectivity that can take values between 0 (response to any odour) and 1 (response to only one odour) [29]. Next, we wanted to know whether these neurons would be even more selective when tested at lower odour concentrations, so we performed dose–response experiments with the best ligands. The two enantiomers of linalool were tested separately to detect a possible enantioselective response. We found that at a threshold dilution of 10^−4^, A-neuron firing increased only in the presence of (+)-linalool, whereas (−)-linalool, α-terpineol and (*Z*)-jasmone (which have similar vapour pressures to (+)-linalool) were not active at this low odour concentration (figure 1*c*). However, at higher doses, the neuron also responded to (−)-linalool, although to a lesser extent. This response was due to a 2% impurity of the opposite enantiomer (electronic supplementary material, figure S4), which inevitably occurs during synthesis. For example, at a dilution of 10^−2^, the amount of (+)-linalool (1 µg) present in the (−)-linalool stimulus is equivalent to a stimulation with (+)-linalool at a dilution between 10^−3^ (5 µg) and 10^−4^ (0.5 µg). The observed response of A-neurons to higher doses of (−)-linalool could therefore be explained by the low contamination with (+)-linalool. Taken together, our results show a clear enantioselective tuning of A-neurons in female long trichoids to (+)-linalool.

At a 10^−5^ dilution, only benzaldehyde activated B-neurons, whereas the threshold dilution for isovaleric acid was 10^−4^. The other two best ligands, benzoic acid and valeric acid, did not elicit a response below a dilution of 10^−3^ (figure 1*c*). However, benzoic acid has a vapour pressure a thousand times lower than the other three odours, which are comparable in volatility. This means that the probability of benzoic acid molecules reaching the antenna is much lower than for the other stimuli. Our results thus confirmed benzoic acid as one of the best ligands for B-neurons [7], and showed that isovaleric acid is similarly active to benzaldehyde.

### (b) Receptive range of medium sensilla trichodea

The two OSNs in the second physiological type of sensilla trichodea regularly had very similar spike amplitudes (figure 1*d*). We referred to this type as medium trichoids, which have been described to often house OSNs of similar diameter and consequently similar spike amplitudes [5,30]. As spike sorting was therefore difficult, we analysed the pooled response of both neurons (figure 1*e*). Interestingly, the four best ligands α-terpineol, (*Z*)-jasmone, methyl salicylate and 2-phenyl ethanol were also ligands of A-neurons in long trichoids. A further five odours, including (±)-linalool, were shared between OSNs in medium trichoids and A-neurons in long trichoids. The remaining seven A-neuron ligands did not elicit a response from medium trichoids. The sesquiterpene germacrene D and the headspace of mulberry leaves (table 1) were minor but specific ligands for medium trichoids. As with long trichoids, bombykol and bombykal did not elicit a response. The tuning width of OSNs in medium trichoids (*S* = 0.87) was in the same range as that of OSNs in long trichoids (electronic supplementary material, figure S3*b*). Dose–response experiments using the four best ligands and both enantiomers of linalool showed a tenfold higher threshold dose for medium trichoids (10^−3^; figure 1*f*) than for long trichoids (10^−4^; figure 1*c*). However, OSNs in medium trichoids were again enantioselective as they were more sensitive to stimulation with (+)-linalool. This enantiomer elicited a response at a dilution of 10^−3^, whereas for (−)-linalool the threshold dose was at a dilution of 10^−2^ (figure 1*f*). Although not completely overlapping, OSNs in medium trichoids and A-neurons in long trichoids had a similar response profile, particularly with respect to their enantioselective response to (+)-linalool.

### (c) Response spectra of wild and domesticated silkmoths are similar

*Bombyx mori* silkmoths have been bred under human care for more than 5000 years, resulting in a reduced number of olfactory and gustatory sensilla compared with their extant wild ancestors *B. mandarina* [31,32]. As the receptive range and/or sensitivity of OSNs in trichoids of the domesticated female may also have changed, we tested active ligands from domesticated females in wild females. We found that the overall response profiles of OSNs were very similar between the two species (electronic supplementary material, figure S5*a*). This was particularly true for A- and B-neurons in long trichoids, whereas OSNs in ‘wild’ medium trichoids showed a broader tuning than the corresponding ‘domesticated’ neurons. Furthermore, the same characteristic enantioselective response to (+)-linalool was present in both species for A-neurons in long trichoids and for OSNs in medium trichoids, with the only difference that ‘wild’ neurons were more sensitive to this odour (electronic supplementary material, figure S5*b*). In addition to the reduced number of olfactory sensilla, this attenuation of OSN sensitivity may contribute to the reduced antennal response to stimulation with (±)-linalool in female *B. mori* [32].

### (d) Behavioural relevance of odours detected by sensilla trichodea

**Figure 2.**
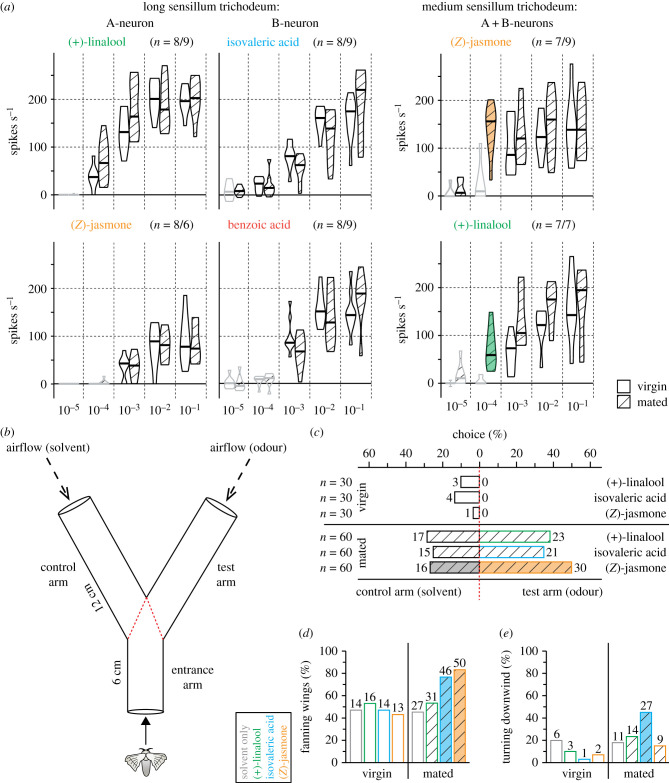
Mating-dependent sensitivity and odour-guided behaviour of female silkmoths. (*a*) Comparison between single sensillum recording responses of virgin and mated females. *Violin plots*, net maximum spike frequencies (spikes s^−1^) to five (v/v) odour concentrations corresponding to 60 ng, 600 ng, 6 µg, 60 µg, 600 µg pure substance on filter paper; *horizontal line*, median; *grey violin plots*, data not different from zero (*p* > 0.05, Wilcoxon rank sum test); *filled violin plots*, data from virgin and mated females differ (*p* ≤ 0.003, Mann–Whitney U-test). (*b*) Experimental setup (Y-maze) with pulsed airstreams. *Test arm*, 10 µl odour (1 : 10^4^); *control arm*, 10 µl solvent; *red dotted line*, virtual decision line. Experiments ended when the female crossed the decision line with its thorax or after 10 min. (*c*) Outcome of Y-maze assay. *Bars*, proportion of females that chose control or test arm; *filled bar*, difference from equal distribution (*p* = 0.039, *χ*^2^ goodness of fit test). (*d*) Proportion of females fanning their wings in entrance arm. *Filled bars*, difference from tests with solvent only (*p* ≤ 0.0007, Fisher's exact test with Bonferroni–Holm correction). (*e*) Proportion of females turning downwind in entrance arm. *Filled bar*, difference from tests with solvent only (*p* = 0.003, Fisher's exact test with Bonferroni–Holm correction). Absolute numbers next to bars in (*c*–*e*).

Neuronal sensitivity could be increased after mating if OSNs were critical for host plant location. We found that only OSNs in medium trichoids were more sensitive in mated females than in virgin females (figure 2*a*), demonstrating that medium trichoids may be involved in the selection of oviposition sites. We next established an assay to test the odour-evoked behaviour of individual females to the major ligands of sensilla trichodea. Since pulses of pheromone filaments are more efficient at attracting male moths than a continuous stream of pheromone [33], we constructed a Y-maze with pulsed olfactory stimulation (figure 2*b*), and tested this setup with male silkmoths and their response to bombykol (1 : 10^4^). Out of 30 males tested, 29 made a choice, with 27 males choosing the pheromone arm and 2 males choosing the control arm (*p* < 0.0001, *χ*^2^ goodness of fit test). Having confirmed that our assay was suitable for studying odour-guided behaviour in silkmoths, we tested virgin and mated females with (+)-linalool, isovaleric acid or (*Z*)-jasmone versus solvent (figure 2*c*). Regardless of the odour tested, virgin females rarely entered the test or control arm (3–13% for each odour), and none of those that made a choice chose the test arm. Interestingly, mating had a strong effect on silkmoths' motivation to move, with around two-thirds of mated females crossing the decision line (*p* < 0.0001 for each of the three odours, Fisher's exact test). When tested with (+)-linalool or isovaleric acid, females randomly chose one of the arms of the Y-maze. However, in experiments with (*Z*)-jasmone, females preferred the test arm, demonstrating that (*Z*)-jasmone is an attractive signal for mated female silkmoths.

While in the entrance arm, females sometimes fanned their wings intensely or turned 180° downwind. These behaviours could be related to attraction (wing fanning) or aversion (downwind turning). To test whether the frequency of these behaviours was dependent on the presence of odour, we performed additional tests with virgin (*n* = 30) and mated (*n* = 60) females, where both arms of the Y-maze were control (solvent) arms. About half of the virgin females fanned their wings, regardless of whether an odour was present or not. However, after mating, the attractive (*Z*)-jasmone and the supposedly neutral isovaleric acid increased the proportion of wing-fanning females to about 80% (figure 2*d*). We then counted the number of females that turned 180° downwind. This behaviour was rarely observed in virgin females and was independent of the presence or absence of odour. Remarkably, a higher proportion of mated females turned downwind in tests with isovaleric acid—but not with (+)-linalool or (*Z*)-jasmone—than in tests with solvent alone (figure 2*e*). These results suggest that odour-induced wing fanning in mated females may be a sign of both attraction (to (*Z*)-jasmone) and aversion (away from isovaleric acid).

### (e) Non-canonical expression of olfactory receptors

To elucidate the molecular basis of the observed response spectra, we examined the expression of five ORs with a known female bias in the antenna [27], the (*Z*)-jasmone receptor *BmorOr56* [9] and *ORco*. As the acid response of B-neurons in long trichoids indicates the expression of acid-sensing IRs, we also investigated the presence of the corresponding IR-co-receptor *Ir8a* [20]. We qualitatively assessed the presence of each receptor and, where possible, assigned the receptor to a sensillum type (table 2).

**Table 2.** Expression of candidate ORs, *ORco* and *Ir8a* in the female antenna*.*

receptor	frequency	long trichoids	medium trichoids	sensilla basiconica	sensilla coeloconica
*ORco*	++++++++++	x	x	x	−
*BmorOr19*	+++++	x	x	−	−
*BmorOr45*	+++++	x	−	−	−
*BmorOr47*	+++++	x	−	−	−
*BmorIr8a*	++++	x	−	−	x
*BmorOr12*	++	−	−	x	−
*BmorOr56*	++	?	?	?	−
*BmorOr30*	+	x	x	−	−

**Figure 3.**
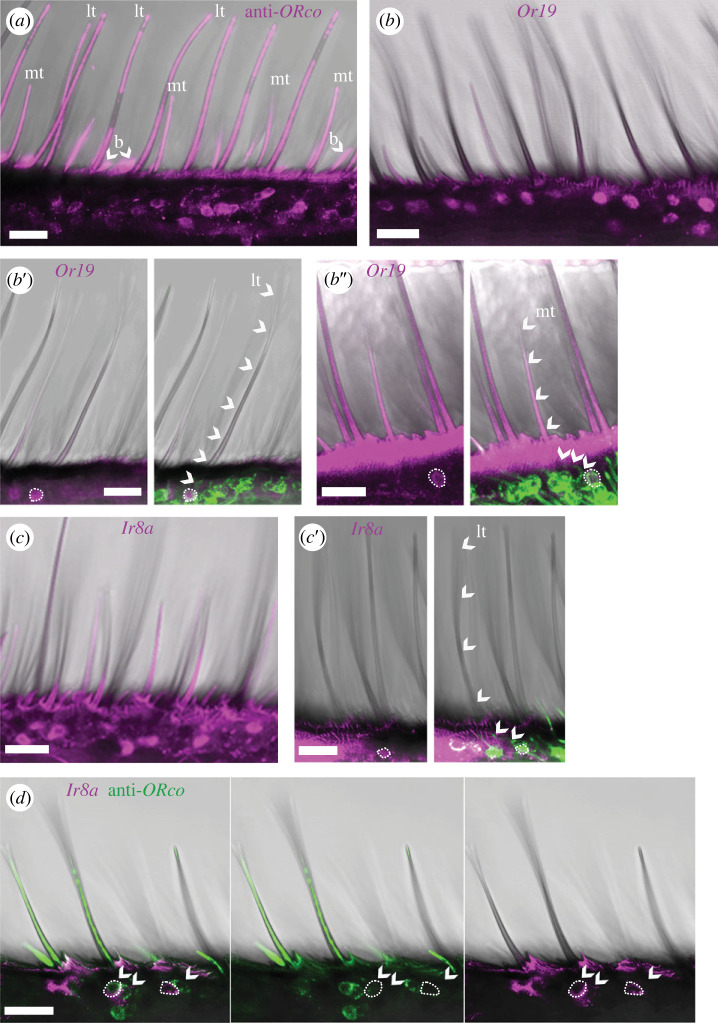
Expression of olfactory receptors in the female antenna. Fluorescent RNA *in situ* hybridization (FISH) and fluorescent immunohistochemistry (FIHC) alone or in combination. Fluorescence channels overlaid with the transmitted light channel. Scale bars, 20 µm. (*a*) FIHC with anti-*ORco* antibody [34] (magenta) labelling somata and dendrites of OSNs that innervate medium trichoid (mt), long trichoid (lt) and basiconic (b) sensilla. (*b*) FISH with *Or19*-specific RNA probe (magenta) combined in (*b*’,*b*’’) with FIHC using anti-HRP antibody (green, neuronal marker), showing innervation (arrowheads) of long trichoids (*b*’) and medium trichoids (*b*’’) by *Or19*-expressing neurons (encircled somata). (*c*) FISH with *Ir8a*-specific RNA probe (magenta) combined in (*c*’) with FIHC using anti-HRP antibody (green), showing innervation (arrowheads) of a long trichoid by an *Ir8a*-expressing neuron (encircled soma). (*d*) FISH with *Ir8a*-specific RNA probe (magenta) combined with FIHC using anti-*ORco* antibody (green). Encircled, *Ir8a*-positive somata; arrowheads, *ORco*-positive somata.

As expected, *ORco* had by far the highest expression in the female antenna and was present in sensilla trichodea and basiconica (figure 3*a*) but not in sensilla coeloconica (electronic supplementary material, figure S6*a*). *BmorOr19* was also highly abundant (figure 3*b*) and expressed by neurons in long trichoids (figure 3*b*’). Interestingly, *BmorOr19* was also expressed by neurons associated with medium trichoids (figure 3*b*’’). *BmorOr45* and *47* had similarly high expression (electronic supplementary material, figure S6*b*,*c*) with dendrites in long trichoids (electronic supplementary material, figure S6*b*’,*c*’). The next most frequently labelled receptor was *BmorIr8a* (figure 3*c*), which was associated with neurons located in long trichoids (figure 3*c*’) and in sensilla coeloconica (electronic supplementary material, figure S6*d*). In long trichoids, *BmorIr8a* mainly occurred in cells co-labelled with an antibody against *ORco*, but occasionally we observed a *BmorIr8a*-positive cell without co-expression of *ORco*. Adjacent to a *BmorIr8a*-positive cell (with or without *ORco* co-expression), we typically found an *ORco*-positive cell (figure 3*d*).

*BmorOr12* expression in the antenna was sparse and *BmorOr12*-positive cells had dendrites in sensilla basiconica (electronic supplementary material, figure S6*e*). Similarly, *BmorOr56* had a sparse expression pattern (electronic supplementary material, figure S6*f*). However, we could not assign *BmorOr56* to any sensillum type. *BmorOr30* was rarely detected in the antenna (electronic supplementary material, figure S6*g*) but was present in both long and medium trichoids (electronic supplementary material, figure S6*g*’,*g*’’), as was *BmorOr19*. Owing to its low expression, *BmorOr30* may have only a small contribution to the response profile of sensilla trichodea.

## 3. Discussion

We investigated the biological role of long and medium sensilla trichodea, the most frequent sensillum types on the antenna of female *B. mori*. Since A-neurons in long trichoids respond to the mulberry component (±)-linalool [2], it has been postulated that female A-neurons may be responsible for host plant detection [8,28]. However, as none of the OSNs in long trichoids responded to the scent of mulberry leaves (table 1), this hypothesis does not seem to be correct. In the same line, long trichoids of female *M. sexta* do not respond to the mixture of volatiles released by leaves of typical host plants [35], and host plant bouquets do not activate regions in the first olfactory processing centre in the brain of *M. sexta* that are presumably targeted by OSNs housed in long trichoids [36]. Taken together, these and our results do not support the hypothesis that A-neurons in long trichoids of female moths are involved in oviposition behaviour.

We found that A-neurons responded with high sensitivity only to (+)-linalool (figure 1*c*). Enantiomer-specific physiological or behavioural responses to linalool are known from females of other moth species such as *M. sexta* [37], *Mamestra brassicae* [38] and *Trichoplusia ni* [39]. Examples of sensitive, enantioselective and narrowly tuned receptors have typically been found among pheromone receptors [40], suggesting that A-neurons in female long trichoids may be involved in pheromone communication, comparable to sex pheromone-detecting A-neurons in male long trichoids [6]. This hypothesis raises the question of whether (+)-linalool might be a male-produced pheromone, as in *T. ni*. Males of this species emit a pheromone mixture that is attractive to females and consists of more than 80% (+)-linalool [39]. Accordingly, female *T. ni* have trichoids that are specifically tuned to (+)-linalool [41]. Male pheromones are typically released from eversible, male-specific scales (hair pencils) on the abdomen of many moth species [42]. Although *B. mori* males also possess tiny hair pencils, no volatile compounds have been found to emanate from these scales [28]. In our study, we were able to show that the headspace emanating from the body of live silkmoths contained numerous compounds, including many terpenes (electronic supplementary material, figure S2*b*). However, neither male-specific odours nor linalool were present, and OSNs in female trichoids did not respond to silkmoth body odour (table 1). *Trichoplusia ni* males release higher levels of pheromones when exposed to the female sex pheromone together with the odour of a host plant [43]. Thus, collecting the headspace of *B. mori* males under similar conditions—exposure to bombykol and the odour of mulberry leaves—could increase the emission rate of a putative male pheromone above the neuronal detection limit. In this context, it is noteworthy that volatiles emitted by a single male silkmoth were able to attract some virgin females in a Y-maze (electronic supplementary material, figure S7), whereas (+)-linalool alone did not attract any female (figure 2*c*). Therefore, a potential male sex pheromone could contain a mixture of (+)-linalool and other odours that are only behaviourally active when presented together. However, the reliable and strong attraction of male silkmoths to bombykol [14] is not matched by a similar response of females to (+)-linalool.

In addition to the known ligands for B-neurons in long trichoids, we identified isovaleric acid as a potent activator (figure 1*c*). When tested in our Y-maze, this compound appeared to induce downwind movement (figure 2*d,e*), a behaviour interpreted as olfactory aversion [44]. What might be the ecological significance of this aversion to acids? In *M. sexta*, hexanoic acid and 3-methylvaleric acid are present in the headspace of conspecific larval faeces. Each of these acids alone is capable of deterring females from a host plant to avoid larval competition [13]. Similar results have been reported for noctuid moths [45,46]. In our study, an acid released from silkworm faeces was also repellent to mated silkmoth females and could therefore deter them from crowded oviposition sites.

OSNs co-located in a sensillum often convey information of opposite valence in the same behavioural context, with A-neurons having a positive meaning and mediating approach behaviour, whereas B-neurons often have a negative meaning, leading to aversive behaviour in many insect species [47]. For example, in *B. mori* males, A-neurons detect the attractive sex pheromone bombykol, whereas B-neurons respond to the sex pheromone of other bombycid moth species (bombykal), which acts as a behavioural antagonist [6,48]. It is tempting to assume that the same principle applies to female long trichoids. However, although the best ligand for A-neurons in female long trichoids was behaviourally neutral, one of the best ligands for B-neurons produced a negative signal, consistent with the valence opponency hypothesis.

Response profiles of OSNs housed in medium trichoids and A-neurons in long trichoids were largely overlapping (figure 1*b*,*e*). In particular, neurons in both sensillum types showed a characteristic, enantioselective response to (+)-linalool, suggesting a common molecular basis for these similar olfactory responses. However, OSNs in medium trichoids, but not those in long trichoids, responded to mulberry leaf headspace (table 1), revealing the expression of at least one additional OR specific to neurons in medium trichoids. Among the compounds emitted by mulberry leaves that activated female trichoids, only the sesquiterpene germacrene D was detected by OSNs present in medium trichoids but not in long trichoids, suggesting that silkmoths possess a specialized receptor for germacrene D. Interestingly, about 80% of the OSNs tested in female *Heliothis virescens* responded with high selectivity and sensitivity to germacrene D [49], and OSNs with an identical receptive range were found in two other noctuid species [50]. Germacrene D therefore appears to be an important plant-related olfactory signal in moths belonging to taxonomically distant families, and could be detected by related and so far unknown ORs.

In addition to the response to mulberry, only OSNs in medium trichoids showed increased sensitivity after mating (figure 2*a*), further supporting the hypothesis that they may play a role in the context of oviposition. In a Y-maze, mated females were attracted to the mulberry scent (*Z*)-jasmone at a dilution of 10^−4^. At this dose, OSNs in medium trichoids of mated females appeared saturated (median response of 156 spikes s^−1^), whereas A-neurons in long trichoids did not respond at all to (*Z*)-jasmone (figure 2*a*). Therefore, the attraction of mated females to (*Z*)-jasmone could be attributed to the activation of OSNs in medium trichoids, although putative (*Z*)-jasmone-sensing OSNs in other sensillum types may also be involved.

**Figure 4.**
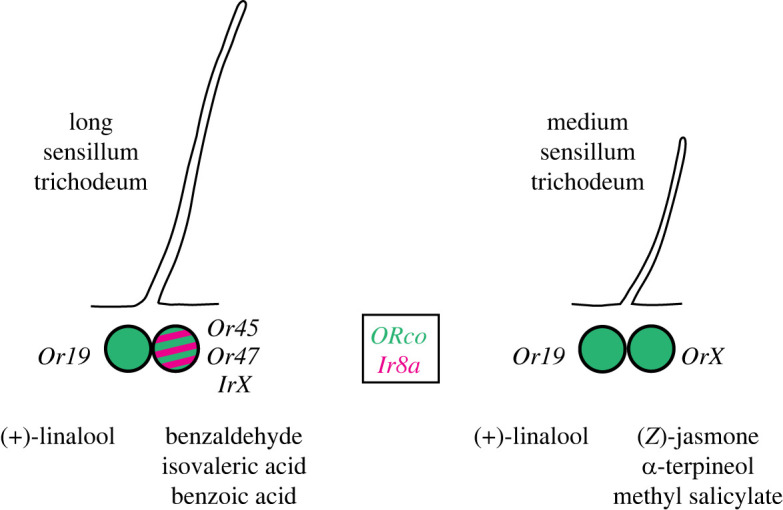
Proposed expression of olfactory receptors and their best ligands in female sensilla trichodea. *Green circle*, soma of *ORco*-positive OSN, *green circle with magenta stripes*, soma of *ORco/IR8a*-positive OSN. *IrX*, *OrX*, unidentified IRs or ORs.

Several response characteristics of sensilla trichodea of female silkmoths suggest a non-canonical expression of olfactory receptors (summarized in figure 4). We show that OSNs in long and medium trichoids respond with high sensitivity and selectivity to (+)-linalool (figure 1*c*,*f*). This is reminiscent of the response to the same given pheromone component in different trichoid sensillum types of male moths described for *B. mori* [5], *M. sexta* [51], *H. virescens* and *Antheraea* spp. [52,53]. Other insects such as tsetse flies [54] or ambrosia beetles [55] have also been found to have OSNs housed in different sensillum types but with highly correlated response profiles. These observations suggest that a given OR may be expressed by OSNs located in different sensillum types and that these OSNs may therefore co-localize with different OSNs. This expression pattern differs from the fixed pairing paradigm of OSNs in the periphery shown in *D. melanogaster* [16,23,56]. In our study, we found that OSNs in both long and medium trichoids of *B. mori* females do indeed express the linalool-detecting *BmorOr19* (figure 3*b*) and most likely co-localize with neurons expressing a different receptor depending on the sensillum type. We were thus able to reveal the molecular basis of physiological results previously obtained in a wide range of insect species, indicating a consistent violation of the stereotypical pairing rule of OSNs established in *D. melanogaster*.

Since B-neurons in long trichoids respond to acids (figure 1), we hypothesized that B-neurons might express *Ir8a*, the co-receptor of acid-sensing IRs in several insects such as vinegar flies, mosquitoes and moths [13,20,57,58]. Indeed, we found that *BmorIr8a* was highly expressed in neurons in female trichoids (figure 3*c*), a sensillum type that typically houses OSNs that express ORs but not IRs [16]. Our result is in agreement with a recent study showing by RT-PCR that *BmorIr8a* is expressed in the antenna of both sexes of *B. mori* [59]. In the same paper, three female-biased IRs with similar expression levels to *BmorIr8a* were described (*BmorIr31a*, *75p.2*, *75q.1*), which could be promising candidates to be expressed with *BmorIr8a* in female long trichoids. In addition, we found that *BmorIr8a* is also abundantly expressed in the antenna of males (electronic supplementary material, figure S8), suggesting that acid sensing may play a prominent role in silkmoths in general. Another example of the presence of *Ir8a* in non-coeloconic sensilla of male and female moths is *Agrotis segetum* [60]. However, in this study it was not possible to decide whether *AsegIr8a* was expressed in sensilla trichodea or basiconica or in both types of sensilla.

A *BmorIr8a*-positive cell was usually found in close proximity to an *ORco*-positive cell, suggesting that both cells were neighbours in the same sensillum, and thus representing an exception to the rule that OR- and IR-expressing neurons are located in mutually exclusive sensillum types [16,20]. Similarly, trichoids on the antenna of the cockroach *Periplaneta americana* house a *PamORco*-positive A-neuron and a *PamORco*-negative B-neuron that responds to acids [61]. Thus, in this distantly related hemimetabolous insect, there is a pairing of OR- and presumably IR-expressing neurons in the same sensillum trichodeum, parallel to our results in *B. mori*. In the silkmoth antenna, however, we found mostly *BmorIr8a*-positive cells that were also *ORco*-positive, indicating co-expression of acid-sensing IRs together with ORs. We also found both types of *Ir8a*-expressing neurons (*Ir8a*-positive or *Ir8a/ORco*-positive) in the male silkmoth antenna. More recently, *ORco* and one or more of the IR-co-receptors *Ir8a*, *Ir25a* and *Ir76b* have been found to be frequently co-expressed in OSNs of vinegar flies [62] and mosquitoes [63]. Furthermore, co-expression of co-receptors could be mapped to specific sensillum types in the fly, revealing co-expression of *ORco*/*Ir8a* in sensilla coeloconica and basiconica, whereas it was absent in sensilla trichodea [62]. In both flies and mosquitoes, *ORco*/*Ir8a* was the rarest of all combinations of co-expressed co-receptors. By contrast, our study shows that *ORco* and *Ir8a* are co-expressed in the most common sensillum type on the antenna of silkmoths.

Taken together, our results suggest a role for medium trichoids of female silkmoths in the detection of host plants for oviposition. The behavioural significance of A-neurons in female long trichoids, which correspond to bombykol-detecting neurons in the male antenna, has not been elucidated, whereas female B-neurons in the same sensillum convey a repellent signal, as in males. While investigating the molecular basis of these observed olfactory responses, we found an unexpected but consistent co-occurrence of *ORco* and *Ir8a* not only in the same sensillum trichodeum but even in a single neuron, and the presence of the female-biased linalool receptor *BmorOr19* in both trichoid sensillum types. These expression patterns extend the receptive range of individual neurons and sensilla and may allow combinatorial olfactory coding already in the periphery.

## 4. Material and methods

Methods regarding headspace collection, WM-FIHC (whole-mount fluorescence immunohistochemistry) and WM-FISH (whole-mount fluorescence *in situ* hybridization) can be found in the electronic supplementary material.

### (a) Animals

*Bombyx mori* (Kinshu x Showa) pupae were purchased from Aseptic Sericulture System Laboratory (Kyoto, Japan) and Laboratorio di sericoltura, Centro di Ricerca Agricoltura e Ambiente (Padova, Italy). *Bombyx mandarina* pupae were provided by Toru Shimada (University of Tokyo, Japan). Male and female pupae were kept in separate boxes at room temperature. After eclosion, moths were stored at 8–10°C until they were used for experiments at days 1 to 8 after eclosion. To obtain mated females, a *B. mori* couple was placed in a small plastic box at room temperature. Copulation usually started immediately and lasted for several hours. After 7 to 13 h, couples were separated and females used for experiments.

### (b) Odorants

Odorants were from Sigma-Aldrich (http://www.sigma-aldrich.com), Chem Faces (http://www.chemfaces.com) and BOC Sciences (https://www.bocsci.com). The enantiomers of linalool were synthesized by Wittko Francke (University of Hamburg, Germany), and bombykal was synthesized from bombykol (Pherobank, https://www.pherobank.com) by Jerrit Weissflog (MPI for Chemical Ecology Jena, Germany). For screening odorants (*n* = 76; electronic supplementary material, table S1) in SSR, odorants were diluted in hexane (10^−2^) or acetone (benzoic acid, 10^−2^); for dose–response experiments, serial dilutions of odorants were made (10^−5^, 10^−4^, 10^−3^, 10^−2^ and 10^−1^). For behavioural assays, odorants were diluted in mineral oil (10^−4^).

### (c) Single sensillum recordings

For SSR, we performed cut-tip single sensillum recordings [64]. The antenna of a female was cut at the base. The glass capillary of the reference electrode filled with haemolymph Ringer (6.4 mmol l^−1^ KCl, 20 mmol l^−1^ KH_2_PO_4_, 12 mmol l^−1^ MgCl_2_, 1 mmol l^−1^ CaCl_2_, 9.6 mmol l^−1^ KOH, 354 mmol l^−1^ glucose, 12 mmol l^−1^ NaCl, pH 6.5) [65] was introduced into the base of the antenna and sealed with Vaseline. As it was impossible to target only one sensillum, several neighbouring sensilla trichodea—long and medium—were cut at the same time with custom-sharpened forceps. Once cut, it was difficult to decide which trichoid subtype was contacted in a given recording. However, the physiological characteristics of both sensillum types were clearly different, so that long and medium trichoids could be distinguished unambiguously. The glass capillary of the recording electrode was filled with sensillum Ringer (171.9 mmol l^−1^ KCl, 9.2 mmol l^−1^ KH_2_PO_4_, 10.8 mmol l^−1^ K_2_HPO_4_, 3 mmol l^−1^ MgCl_2_, 1 mmol l^−1^ CaCl_2_, 1.5 mmol l^−1^ HCl, 22.5 mmol l^−1^ glucose, 25 mmol l^−1^ NaCl, pH 6.5) [65]. The antenna was placed under a microscope, where a PEEK tube, providing a constant, humidified charcoal-filtered air stream (0.5 l min^−1^), was directed to the recording site with outlet at 2 cm distance from antenna. When inserting an odour stimulus (0.4 l min^−1^) into the air stream, it switched automatically to an additional compensatory air flow (Syntech CS-55 Stimulus Controller, https://www.ockenfels-syntech.com). The recording electrode was directed to cover the tip of a cropped sensillum trichodeum. Filter papers loaded with odorants were freshly prepared before each experiment. Six microlitres of diluted odorants, 10 µl of eluted headspace (mulberry leaves, silkmoths), 10 µl diluted meconium or 10 µl silkworm faeces suspension were pipetted on a filter paper placed in a glass pipette. AutoSpike32 (v3.7) measured changes in extracellular potentials. Signals were 10× amplified (Syntech AC/DC probe), sampled with 48 000 Hz, and filtered (300–3 kHz with 50/60 Hz suppression). Neuronal activity was recorded 3 s before and 20 s after the stimulus pulse (duration: 0.5 s). Each sensillum trichodeum type (long or medium) was recorded only once per antenna. We analysed action potential frequency (spikes s^−1^) during the recording interval using a bin width of 25 ms. In long trichoids, two neurons could be differentiated based on their different spike amplitudes, while the two neurons in medium trichoids mostly had similar spike amplitudes. Very rarely, we encountered trichoids showing activity from one or three neurons; these were excluded from the present study. The odour-evoked response of OSNs was quantified by subtracting the maximum spiking frequency during 1 s before stimulus onset from the maximum spiking frequency during 1 s after stimulus onset.

### (d) Behavioural assay

We established a two-choice assay for female silkmoths with each experimental arm of the Y-maze (diameter 2.8 cm, length 12 cm) connected to a 100 ml glass bottle containing 1 ml of the solvent mineral oil (control arm) or 1 ml of the diluted odorant (10^−4^, test arm). To prevent side-biased effects, we switched the position of control and test arm after each experiment. Humidified air was pulsed for 2 s (interstimulus interval 2 s) through the glass bottles into the experimental arms of the Y-maze (0.3 l min^−1^). Air was pulled out through the entrance arm (diameter 2.8 cm, length 6 cm) of the Y-maze at 0.9 l min^−1^ to ensure the odour flow through the setup. A camera mounted above the setup recorded the moth's behaviour. Silkmoths were placed in the experimental room (25°C, 70% relative humidity) 30 min before testing. Experiments started at the end of the photo phase (12 h light : 12 h dark). A single moth was placed in the entrance arm of the Y-maze and was observed until it crossed a virtual decision line with its thorax, or until the end of the experiment after 10 min, respectively.

### (e) Statistical analysis

Sample sizes and statistical tests used are given in the text and figure legends. Statistical tests were performed using GraphPad InStat (v3.10) and Social Science Statistics (https://www.socscistatistics.com/).

All data related to figures 1 and 2, electronic supplementary material, figure S5 and table 1 can be found in Raw data file.xlsx in the electronic supplementary material.

## Data Availability

Supplementary material is available online [66].
